# Complicated Intra-Abdominal Infections Observational European study (CIAO Study)

**DOI:** 10.1186/1749-7922-6-40

**Published:** 2011-12-09

**Authors:** Massimo Sartelli, Fausto Catena, Luca Ansaloni, Daniel V Lazzareschi, Korhan Taviloglu, Harry Van Goor, Pierluigi Viale, Ari Leppaniemi, Carlo De Werra

**Affiliations:** 1 Department of Surgery, Macerata Hospital, Italy; 2 Department of General and Transplant surgery, St Orsola-Malpighi University Hospital, Bologna, Italy; 3 Department of Surgery, "Ospedali Riuniti" Hospital, Bergamo, Italy; 4 Department of Neuroscience, UT Southwestern Medial Center, USA; 5 Department of Surgery, University of Istanbul, Turkey; 6 Radboud University Nijmegen Medical Centre, The Netherlands; 7 Clinic of Infectious Diseases, Department of Internal Medicine Geriatrics and Nephrologic Diseases, St Orsola-Malpighi University Hospital, Bologna, Italy; 8 Department of Surgery, Helsinki University Central Hospital, Finland; 9 Department of General Surgery, University of Naples "Federico II", Italy

## Abstract

Complicated intra-abdominal infections are frequently associated with poor prognoses and high morbidity and mortality rates.

Despite advances in diagnosis, surgery, and antimicrobial therapy, mortality rates associated with complicated intra-abdominal infections remain exceedingly high.

In order to describe the clinical, microbiological, and management-related profiles of both community-acquired and healthcare-acquired complicated intra-abdominal infections (IAIs), the World Society of Emergency Surgery (WSES), in collaboration with the Surgical Infections Society of Europe (SIS-E) and other prominent European surgical societies, has designed the CIAO study.

The CIAO study is a multicenter, observational study and will be carried out in various surgical departments throughout Europe. The study will include patients undergoing surgery or interventional drainage for complicated IAI.

## Background

Intra-abdominal infections (IAIs) include a wide array of pathological conditions, ranging from uncomplicated appendicitis to fecal peritonitis.

From a clinical perspective, IAIs are classified in two distinct groups: uncomplicated and complicated infections [1].

In uncomplicated IAIs, the infectious process involves only a single organ and does not extend to the peritoneum. Patients with uncomplicated infections can be treated surgically by means of resection or non-operatively with antibiotic therapy. When the focus of infection is effectively treated by surgical excision, 24-hour perioperative prophylaxis is typically sufficient. Patients with intra-abdominal infections, including acute diverticulitis and certain forms of acute appendicitis, may be managed non-operatively.

In complicated IAIs, the infectious process extends beyond a singly affected organ, and causes either localized peritonitis (intra-abdominal abscess), or diffuse peritonitis. The treatment of patients with complicated intra-abdominal infections involves both source control and antibiotic therapy.

Intra-abdominal infections are further classified as either community-acquired intra-abdominal infections (CA-IAIs) or healthcare-associated intra-abdominal infections (HA-IAIs). CA-IAIs, as the name implies, are acquired directly in the community while HA-IAIs develop in hospitalized patients or residents of long-term healthcare facilities. Of the two, the latter is associated with higher rates of mortality due to the patients' poorer underlying health and an increased likelihood of infection by multi-drug resistant microorganisms [2].

Source control encompasses all measures undertaken to eliminate the source of infection and control ongoing contamination [3].

The appendix is the most common source of infection in community-acquired intra-abdominal infections, followed closely by the colon and stomach. Dehiscences complicate 5-10% of intra-abdominal bowel anastomoses, and are associated with increased mortality rates [4].

Control of the septic source can be achieved by both operative and non-operative means.

Non-operative interventional procedures involve the percutaneous drainage of abscesses.

Ultrasound- and CT-guided percutaneous drainage of abdominal and extra-peritoneal abscesses have proven to be safe and effective in select patients [5-12].

Surgery is the most important therapeutic recourse for controlling intra-abdominal infections.

Patients suffering from severe peritonitis are prone to persisting intra-abdominal infection, even when the source of infection has been neutralized. In these cases, timely re-laparotomy is the only surgical recourse known to significantly improve patient outcome.

Additionally, it should be pointed out that a single procedure may not suffice, and further surgical exploration may be necessary to achieve adequate source control [13-16].

In the event of secondary peritonitis, deciding whether a re-laparotomy is the proper course of action, and if so, when the procedure should be performed, is largely subjective and often based on a surgeon's professional experience. Factors indicative of progressive or persistent organ failure during early postoperative follow-up analysis are the strongest indicators of ongoing infection and suggest positive findings upon re-laparotomy [17-19].

Three methods of localized, mechanical management of abdominal sepsis following the initial laparotomy, which was performed for purposes of source control, are currently debated within the medical community:

(1) Open-abdomen

(2) Planned re-laparotomy,

(3) On-demand re-laparotomy

In 2007, van Ruler et al. [20] published the findings of a randomized, clinical trial comparing on-demand and planned re-laparotomies for patients with severe peritonitis.

During the course of the trial, a total of 232 patients with severe intra-abdominal infections (116 planned and 116 on-demand) were randomized.

In the planned re-laparotomy group, re-laparotomies were performed every 36 to 48 hours following the index laparotomy to inspect, drain, lavage, and perform other necessary abdominal interventions for residual peritonitis or newly established focal infections.

In the on-demand re-laparotomy group, re-laparotomies were only performed on those patients demonstrating clinical deterioration or lack of clinical improvement due to intra-abdominal pathology.

Patients in the on-demand re-laparotomy group failed to demonstrate a statistically significant decrease in the rate of adverse treatment outcomes compared to patients in the planned re-laparotomy group, but these patients did feature a substantial reduction in re-laparotomies, general health care utilization, and overall medical costs.

Antimicrobial therapy also plays an integral role in the management of intra-abdominal infections; indeed, to ensure optimal patient outcome, empiric antibiotic therapy should be initiated as early as possible. The misuse of antibiotic regimens (by administering inappropriate antimicrobial agents, for example), is perhaps the strongest predictor of unfavorable treatment outcome [21-24].

The initial antibiotic therapy for IAIs is usually empiric given that the patient is often critically ill and microbiological data (culture and susceptibility results) can take a minimum of 48 hours to become available.

Empiric antibiotic therapy considers the most frequently isolated germs as well as any local trends of antibiotic resistance.

The major pathogens involved in community-acquired intra-abdominal infections are *Enterobacteriaceae *and anaerobic microbes (especially *B. fragilis*).

Bacterial drug resistance has become a very serious problem, particularly given that rates of antimicrobial resistance continue to increase despite a distinct lack of new antimicrobial agents currently in development.

In the last decade, the emergence of multidrug-resistant (MDR) bacteria, such as extended-spectrum beta-lactamase (ESBL)-producing Enterobacteriaceae, *Pseudomonas aeruginosa, Acinetobacter baumannii*, Vancomycin-resistant *Enterococcus*, and Methicillin-resistant *Staphylococcus aureus*, has become a pressing issue in the treatment of intra-abdominal infections.

The increasing emergence of multidrug-resistant bacteria combined with a scant pipeline of new antibiotics to combat these infections (which is particularly disconcerting for infections by gram-negative microorganisms) has been documented in a recent report by the European Antimicrobial Resistance Surveillance System [25].

In the specific context of intra-abdominal infections, the main resistance problem is posed by ESBL-producing Enterobacteriaceae, which are commonly identified in community-acquired infections.

The recent and rapid spread of carbapenemases in *Klebsiella pneumoniae *(KPC) has become an important concern when administering antimicrobial therapy in hospitals worldwide. Scrupulous optimization of the use of carbapenems based on indication and exposure is of utmost importance [26].

Samples obtained from intra-abdominal surgery or interventional drainage procedures should be cultured; these samples should be of sufficient volume (at least 1 mL of fluid or tissue, preferably more) and should be sent to the laboratory for detailed analysis using an appropriate transport system.

## Methods

### Aim

The purpose of the study is to describe the clinical, microbiological, and treatment profiles of community-acquired and healthcare-acquired complicated intra-abdominal infections (IAIs) in Europe.

### Study population

This prospective multicenter observational study will be performed in various European medical institutions over a 6-month period (January-June 2012). Patients undergoing surgery or interventional drainage to address complicated IAI, or patients who have yieded positive microbiological cultures upon postoperative drainage (intra-abdominal samples taken from surgery or drainage) will be included in the database. Patients with pancreatitis, primary peritonitis from cirrhosis, or ascites will not be included in the study.

### Study design

This observational study will not attempt to change or modify the laboratory or clinical practices of the participating physicians, and neither informed consent nor formal approval by an Ethics Committee will be required.

The study will meet and abide by the standards outlined in the Declaration of Helsinki and Good Epidemiological Practices.

### Data collection

In each center, the coordinator will collect and compile data in an online case report system.

These data will include the following: (i) patient and disease characteristics, i.e., demographic data, type of infection (community- or healthcare-acquired), severity criteria, previous curative antibiotic therapy administerd in the 7 days preceding surgery; (ii) origin of infection and surgical procedures performed; and (iii) microbiological data, i.e., identification of bacteria and microorganismal pathogens within the peritoneal fluid, the presence of yeasts (if applicable), and the antibiotic susceptibilities of bacterial isolates.

### Statistical analysis

Following data entry into a computerized database, the results will be expressed as standard statistical metrics: median (range), mean ± standard deviation for continuous variables, and the number of patients (with the corresponding percentages) for other qualitative variables. The primary endpoints will include

• Clinical profiles of intra-abdominal infections

• Epidemiological profiles (the epidemiology of the microorganisms isolated from intra-abdominal samples and these organisms' resistance to antibiotics)

• Management profiles

Comparisons will be performed using the Student's *t*-test, *χ*^2 ^analysis, or the Kruskall-Wallis/Wilcoxon tests, as dictated by the natural parameters of the data in question. Statistical significance will be defined as a P-value less than 0.05 (*P *< 0.05). Multivariate analysis will be carried out by means of stepwise logistic regressions in order to assess the predictive factors of mortality during hospitalization. Adjusted odds ratios (OR) and their 95% confidence intervals (CI) will also be included.

### Inclusion Criteria

Patients undergoing surgery or interventional drainage to address complicated IAI, or patients who have yieded positive microbiological cultures upon postoperative drainage (intra-abdominal samples taken from surgery or drainage) will be included.

### Exclusion Criteria

Patients with pancreatitis and primary peritonitis will be excluded.

## Competing interests

The authors declare that they have no competing interests.

## Authors' contributions

MS wrote the manuscript. All authors read and approved the final manuscript.
